# Lactate metabolism and protein lactylation in colorectal cancer: from metabolic reprogramming to epigenetic regulation

**DOI:** 10.3389/fonc.2026.1741782

**Published:** 2026-03-03

**Authors:** Yulan Song, Mingyang Zou, Shaobo Wu, Rongwei Ren, Shundong Yuan, Yixin Pan, Jiebin Pan

**Affiliations:** 1 The Second Hospital & Clinical Medical School, Lanzhou University, Lanzhou, Gansu, China; 2 Department of Orthopaedics, The Second Hospital & Clinical Medical School, Lanzhou University, Lanzhou, Gansu, China; 3 Department of Pathology, The Second Hospital & Clinical Medical School, Lanzhou University, Lanzhou, Gansu, China; 4 Department of General Surgery, The Second Hospital & Clinical Medical School, Lanzhou University, Lanzhou, Gansu, China

**Keywords:** biomarkers, colorectal cancer, immunotherapy, lactate metabolism, lactylation, monocarboxylate transporters, tumor microenvironment

## Abstract

Colorectal cancer (CRC) exhibits profound metabolic reprogramming, in which excessive lactate accumulation remodels the tumor microenvironment and promotes immune suppression, angiogenesis, and therapeutic resistance. Recent studies reveal that lactate also serves as a substrate for lysine lactylation (Kla), linking metabolic overflow to epigenetic regulation. This review focuses on CRC but also incorporates mechanistic data from other tumor models when CRC-specific evidence is limited, synthesizing lactate metabolism, transport, and lactylation into a unified lactate–lactylation axis. Mechanistic analyses highlight the roles of glycolytic enzymes, monocarboxylate transporters (MCT1/4–CD147), and Kla writers, erasers, and readers in driving malignant progression. Based on these insights, a three-step therapeutic framework is proposed: lowering lactate production, blocking lactate shuttling, and restraining Kla-mediated transcriptional reprogramming. Biomarker-guided evaluation using serum lactate dehydrogenase (LDH), tissue Kla immunohistochemistry, and hyperpolarized [1-^13C]-pyruvate MRI provides translational feasibility. This axis offers a mechanistic basis and actionable targets for metabolism-driven precision therapy, particularly in microsatellite-stable CRC (MSS CRC).

## 1 Introduction

Colorectal cancer (CRC) is a leading global malignancy, with ~1.9 million new cases and 900,000 deaths in 2020; it ranks third in incidence and second in cancer mortality worldwide (1). Rates vary by region—higher in Western Europe, Australia/New Zealand, and North America, and lower in Africa and South-Central Asia (2).

Therapeutic options for CRC span surgery, systemic chemotherapy, radiotherapy, and targeted/immunotherapy. Curative-intent resection (with adjuvant therapy when indicated) remains the most common and effective approach for early-stage disease (3). For advanced disease, first-line systemic therapy typically uses oxaliplatin- or irinotecan-based doublets (FOLFOX, FOLFIRI), with escalation to FOLFOXIRI in selected fit patients (4, 5). Radiotherapy is integral to rectal cancer management in the neoadjuvant setting, and immune checkpoint inhibitors are transformative for mismatch repair-deficient/microsatellite instability-high (dMMR/MSI-H) tumors (6, 7). However, despite these advances, long-term control remains challenging due to cumulative toxicity, primary or acquired resistance, and the limited benefit of immunotherapy in microsatellite-stable (MSS) CRC (8). Consequently, the five-year survival for metastatic CRC remains below 15% (9), highlighting an urgent need for new strategies.

CRC exhibits metabolic reprogramming, notably the Warburg effect, which drives aerobic glycolysis and lactate accumulation even under normoxia (10). In CRC, lactate levels reach 5–10 mM; however, CRC exhibits marked metabolic heterogeneity, with subsets of cells retaining substantial mitochondrial oxidative capacity and relying on oxidative phosphorylation alongside glycolysis (11, 12). Lactate is a signaling metabolite and immunomodulator in the tumor microenvironment (TME), promoting invasion, metastasis, angiogenesis, and extracellular matrix remodeling, while suppressing cytotoxic T cells, fostering regulatory T cells, and enhancing myeloid-derived suppressor cells (13, 14). These effects fuel progression, therapy resistance, and poor outcomes.

Beyond serving as a metabolic by-product, lactate also drives epigenetic regulation through lysine lactylation (Kla), first described on histones as a +72 Da modification that links glycolytic overflow to chromatin remodeling and transcription (15). Histone lactylation activates programs associated with immune tolerance, stress adaptation, and tumor progression. In CRC, lactate-induced H3K18la/H4K12la engages writer–eraser–reader circuits to activate programs for immune tolerance, stress adaptation, EMT and angiogenesis; functionally, it sustains stemness, confers ferroptosis resistance, and underlies chemotherapy and anti-VEGF adaptation (16). Complementing chromatin control, non-histone lactylation extends to signaling and effector proteins, thereby tuning translation, DNA-repair fidelity and oncogenic transcription (17).

**Figure 1:**
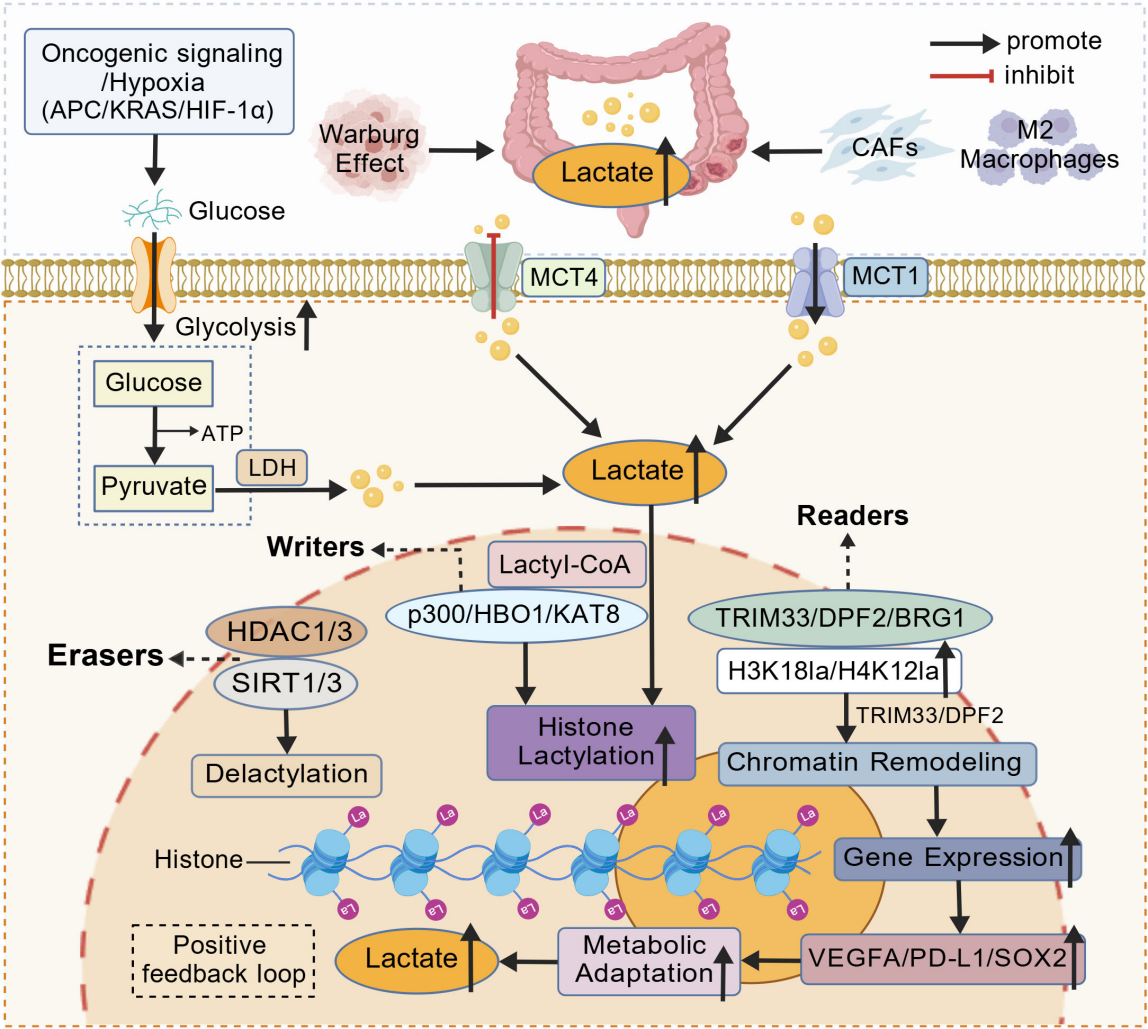
The lactate–lactylation axis in CRC progression Oncogenic signaling and hypoxia in CRC—including APC loss, KRAS activation and HIF-1α stabilization—enhance glycolysis and promote conversion of glucose to pyruvate and lactate through LDH (Warburg effect). Lactate further accumulates in the tumor microenvironment via secretion from stromal cancer-associated fibroblasts (CAFs) and M2 macrophages. Extracellular lactate is exported by MCT4 and imported by MCT1, establishing a tumor–stroma lactate shuttle that sustains metabolic symbiosis. Intracellular lactate is converted to lactyl-CoA, which fuels histone lactylation mediated by writers (p300, HBO1, KAT8), counterbalanced by erasers (HDAC1/3, SIRT1/3) and interpreted by readers (TRIM33, DPF2, BRG1), notably at H3K18la and H4K12la. Lactylation-dependent chromatin remodeling activates transcription of adaptive and pro-tumorigenic genes such as VEGFA, PD-L1 and SOX2, promoting metabolic adaptation, angiogenesis and immune evasion. These processes form a positive feedback loop that reinforces glycolysis, lactate accumulation and CRC progression. Created with BioGDP.com. CRC, colorectal cancer; CAFs, cancer-associated fibroblasts; MCT, monocarboxylate transporter; LDH, lactate dehydrogenase; APC, adenomatous polyposis coli; KRAS, Kirsten rat sarcoma viral oncogene; HIF-1α, hypoxia-inducible factor 1 alpha; HBO1, histone acetyltransferase binding to ORC1 (KAT7); KAT8, lysine acetyltransferase 8; HDAC, histone deacetylase; SIRT, sirtuin; TRIM33, tripartite motif-containing protein 33; DPF2, double PHD fingers protein 2; BRG1, Brahma-related gene 1; VEGFA, vascular endothelial growth factor A; PD-L1, programmed death-ligand 1; SOX2, SRY-box transcription factor 2; H3K18la/H4K12la, histone H3 lysine-18 and H4 lysine-12 lactylation.

Given CRC’s high prevalence, therapeutic challenges, and the pivotal role of lactate in tumor metabolism and signaling, elucidating how lactate metabolism and protein lactylation drive disease progression may reveal biomarkers for early detection and targets to overcome resistance. Here, we refer to the coordinated processes of lactate production, transport and Kla as the “lactate–lactylation axis” in CRC. This axis links glycolytic overflow and monocarboxylate transport to downstream chromatin and signaling programs that drive angiogenesis, immune evasion, and therapeutic responses. This review integrates recent metabolic and epigenetic advances to outline the emerging lactate–lactylation axis and its significance for precision diagnosis and therapy in CRC. A conceptual overview of this lactate–lactylation axis in CRC progression is shown in **Figure 1**.

## 2 Lactate metabolism in CRC

### 2.1 Functional roles of lactate accumulation in CRC progression

#### 2.1.1 Tissue remodeling, EMT, invasion, and angiogenesis

Lactate efflux through MCT4 co-transports protons, acidifying the extracellular space and activating matrix-remodelling proteases (e.g., MMPs, cathepsins, urokinase-type plasminogen activator) that facilitate invasion (18, 19). In CRC models, lactate and LDHA activity drive epithelial–mesenchymal transition (EMT) via TGF-β/SMAD and Wnt/β-catenin signaling, stabilising Snail/Twist and β-catenin, repressing E-cadherin, and enhancing vimentin/N-cadherin, migration, and liver metastasis (20–22). Thus, lactate couples metabolic reprogramming to EMT and invasive behaviour.

Concurrently, lactate is pro-angiogenic: it stabilises HIF-1α and induces VEGF and IL-8 in endothelial cells via NF-κB activation, even in the absence of hypoxia (23, 24). In fibroblasts, lactate engages GPR81 to drive fibroblast-to-myofibroblast transition and extracellular-matrix deposition, collectively sustaining vascular remodelling and metastatic dissemination (25).

#### 2.1.2 Immune suppression and immune evasion

Elevated lactate enforces immunosuppression by inhibiting CD8^+^ T-cell proliferation and IL-2/IFN-γ production through disruption of glycolysis and TCR signaling, while extracellular acidification impairs dendritic-cell antigen presentation and co-stimulation (26, 27). At the same time, lactate expands FoxP3^+^ regulatory T cells via HIF-1α–dependent mechanisms and skews macrophages toward M2/TAM phenotypes expressing Arg1, VEGF, and IL-10, thereby reinforcing an immune-suppressive milieu (28). In CRC models, silencing MCT4 or inhibiting LDHA lowers lactate, increases cytotoxic T-cell infiltration, synergises with PD-1/PD-L1 blockade, and partly restores NK-cell cytotoxicity, linking lactate metabolism to the immune-cold TME and limited immunotherapy efficacy (29, 30).

#### 2.1.3 Stemness, epigenetic regulation, and therapy resistance with niche and microbiota interactions

Lactate sustains cancer stem-like cells via MCT1-mediated uptake and oxidative metabolism under glucose limitation, supporting self-renewal and survival in nutrient-poor niches (31). Notably, CRC exhibits marked metabolic plasticity. Beyond aerobic glycolysis, CRC cells can maintain or preferentially engage mitochondrial oxidative phosphorylation and utilize lactate as an oxidative substrate, particularly in well-oxygenated regions or during therapy adaptation (12). Importantly, lactate-driven resistance is not solely dependent on lysine lactylation, as lactate can independently promote resistance via acidification, redox imbalance, and immunometabolic suppression (32). Lactylation likely serves as a downstream epigenetic consolidation layer that stabilizes these metabolically induced states. Epigenetically, lactate fuels histone Kla; promoter lactylation at loci such as GCLC activates antioxidant programs, suppresses ferroptosis, and contributes to oxaliplatin resistance and stemness, with CRC stem-like cells displaying elevated H3K18la/H4K12la and increased expression of SOX2, NANOG and other stemness regulators (15, 33). Inhibiting LDHA or p300 (which can mediate lactylation) reduces histone lactylation and restores chemosensitivity in experimental models (33). Beyond the primary site, tumor-derived lactate shapes metastatic niches—for example, in colorectal liver metastasis it suppresses local NK-cell function and activates hepatic stellate cells, priming the liver microenvironment for colonization—and gut microbiota can modulate intestinal lactate levels and transporter expression; dysbiosis, including enrichment of Fusobacterium nucleatum, may therefore reinforce immunosuppression and metastatic spread (34–36).

#### 2.1.4 Context-dependent and protective roles of lactate

Although this review primarily addresses pro-tumoural functions of lactate in CRC, accumulating evidence indicates that lactate also exerts context-dependent immunoregulatory and tissue-protective effects. Under physiological, near-neutral conditions, exogenous or endothelial-derived lactate can support oxidative metabolism, promote reparative angiogenesis and tissue regeneration, and in some models enhance TCF1^+^ stem-like CD8^+^ T-cell states and antitumour immunity (37, 38). Recent work further defines lactate as a pleiotropic signaling metabolite whose net impact on immune and stromal cells is determined by its concentration, pH, tissue context and subcellular compartmentalization (39, 40). In the gut, microbiota-derived short-chain fatty acids (SCFAs) such as acetate, propionate and butyrate generally exert barrier-protective and anti-inflammatory effects via GPCR signaling and HDAC inhibition, thereby partially counterbalancing lactate-driven immunosuppression in the colonic mucosa (41). Collectively, these nuances argue against indiscriminate systemic lactate depletion and instead argue for biomarker-guided, locally focused targeting of the lactate–lactylation axis in CRC.

#### 2.1.5 Clinical correlations and translational implications

Serum lactate dehydrogenase (LDH) and lactate-related gene signatures associate with advanced stage, metastatic burden, and poor survival (42, 43); elevated LDH-to-albumin ratios predict adverse outcomes after curative resection. Moreover, transcriptomic classifiers of lactate metabolism stratify prognosis and immune infiltration, nominating subsets for metabolic interventions (44). Therapeutically, combinations of LDHA or MCT inhibitors with chemotherapy or immune checkpoint inhibitors are under preclinical/early clinical evaluation, though metabolic redundancy and systemic toxicity remain challenges (45). Therefore, biomarker-guided selection and spatially resolved metabolic profiling will be essential to identify patients most likely to benefit.

### 2.2 Key enzymes and regulators of lactate metabolism in CRC

#### 2.2.1 The LDH axis and glycolytic activation

Lactate metabolism in CRC reflects coordinated oncogenic reprogramming toward aerobic glycolysis (Warburg effect), yielding abundant lactate as metabolite and signal (10). At the core of this program is the LDH axis. LDHA converts pyruvate to lactate with NAD^+^ regeneration; it is overexpressed in CRC and associates with advanced stage, metastasis, chemoresistance, and poor survival (46, 47). Its transcription is driven by c-Myc (promoter binding) and stabilized by HIF-1α under hypoxia (48). Wnt/β-catenin—frequently activated by APC mutations—also upregulates LDHA and PKM2, coupling canonical oncogenesis to glycolytic flux (49, 50). Conversely, LDHB, favoring lactate-to-pyruvate conversion, is often downregulated, further biasing toward lactate accumulation (51).

Upstream nodes amplify this flux and couple it to proliferation. PKM2, frequently overexpressed in CRC, enhances glycolysis and, in the nucleus, partners with β-catenin to transactivate c-Myc and cyclin D1, reinforcing proliferation–metabolism crosstalk (49, 50). HK2 and GLUT1 are upregulated, boosting glucose uptake and phosphorylation, while PFKFB3 elevates fructose-2,6-bisphosphate to accelerate glycolysis and has been linked to angiogenesis and metastasis (52–54). Together, these accelerators increase substrate availability upstream of LDH.

#### 2.2.2 Mitochondrial diversion and post-transcriptional regulation

Mitochondrial gating further locks cells into a glycolytic state. PDK1 phosphorylates and inactivates the pyruvate dehydrogenase complex, shunting pyruvate away from oxidation and toward lactate, thereby sustaining the glycolytic phenotype (55). In CRC, PDK1 upregulation correlates with proliferation, stemness, and metastasis; its inhibition reduces lactate production and impairs tumor growth (56–58). Thus, carbon flow is diverted toward lactate both by cytosolic acceleration and by restricted mitochondrial entry.

RNA-level control fine-tunes enzyme output. METTL3-mediated m6A stabilizes LDHA mRNA, augments glycolysis, and confers 5-fluorouracil resistance (59). MicroRNAs (miR-34a, miR-20) and circular RNAs regulate LDHA/PKM2, linking non-coding RNA networks to lactate metabolism and drug sensitivity (60, 61).

#### 2.2.3 Spatial metabolic heterogeneity and integrative control

Spatial organization integrates these controls within the tumor microenvironment. Hypoxic cores upregulate LDHA and MCT4 to export lactate, whereas oxygenated margins and stroma express MCT1 to import lactate as oxidative fuel, establishing metabolic symbiosis associated with invasiveness and poor prognosis (62–64). This division of labor ensures continuous lactate production and utilization across niches.

In summary, lactate metabolism in CRC is governed by an interconnected network encompassing oncogenic transcription, post-transcriptional regulation, and spatial organization. Targeting LDHA, PKM2, PDK1, and MCT1/4–CD147 offers therapeutic promise, but pathway redundancy and intratumoral heterogeneity demand biomarker-guided, rational combination strategies (65).

### 2.3 Lactate transport and metabolic symbiosis in CRC

#### 2.3.1 Structure and regulation of lactate transport

**Figure 2:**
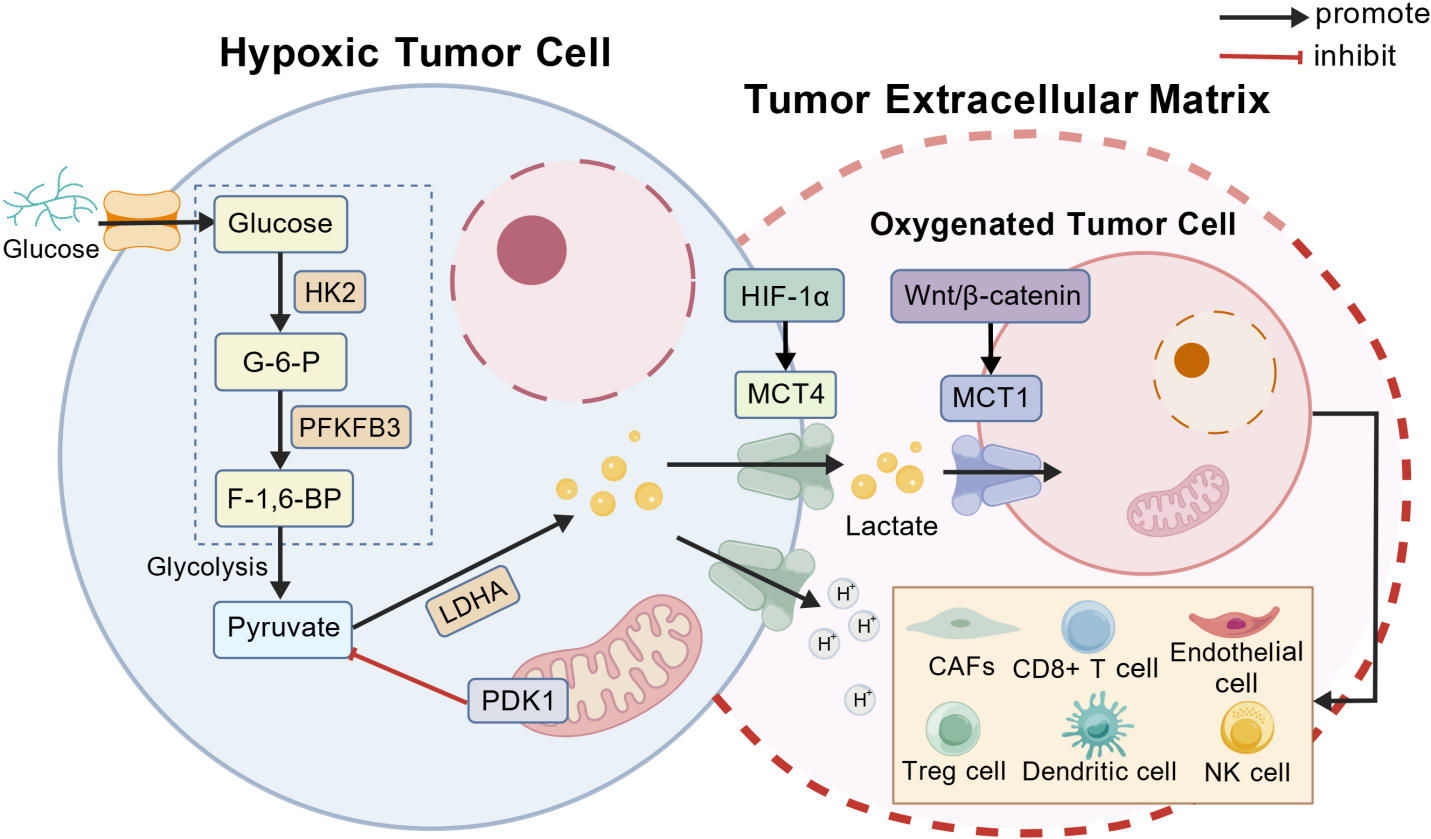
Lactate metabolism and transport symbiosis in CRC. This schematic highlights the conceptual framework rather than exhaustive molecular detail. Under hypoxic conditions, CRC cells enhance glycolysis via key enzymes including HK2, PFKFB3, LDHA, and PDK1, leading to pyruvate conversion into lactate. Stabilized HIF-1α upregulates MCT4 to export lactate and protons from hypoxic tumor cells, while oxygenated tumor cells express MCT1 through Wnt/β-catenin signaling to import lactate as an oxidative substrate. This metabolic coupling forms a lactate shuttle within the tumor microenvironment and supports stromal and immune cell reprogramming. Created with BioGDP.com. CRC, colorectal cancer; HK2, hexokinase 2; PFKFB3, 6-phosphofructo-2-kinase/fructose-2,6-bisphosphatase 3; LDHA, lactate dehydrogenase A; PDK1, pyruvate dehydrogenase kinase 1; HIF-1α, hypoxia-inducible factor-1 alpha; MCT, monocarboxylate transporter; CAFs, cancer-associated fibroblasts; Treg, regulatory T cell; NK, natural killer.

CRC depends on transmembrane lactate flux mediated chiefly by monocarboxylate transporters. MCT4 (SLC16A3), transcriptionally regulated by HIF-1α, is enriched in highly glycolytic, hypoxic cells and exports lactate and protons to prevent intracellular acidification and sustain glycolysis (66, 67). By contrast, MCT1 (SLC16A1) is broadly expressed in oxidative tumor cells and stromal compartments, enabling lactate uptake for tricarboxylic acid (TCA) cycle oxidation (67, 68) (**Figure 2**). This division creates a lactate “shuttle” that supports metabolic symbiosis between tumor subpopulations and between tumor and stroma, enhancing fitness (68). Proper membrane localization and stability of MCT1/MCT4 require the ancillary protein CD147, which also augments MCT4 via HIF-1α–dependent transcription, forming a feed-forward loop that amplifies lactate secretion and symbiosis (69). Co-expression of MCT4 and CD147 correlates with invasion, angiogenesis, and poor prognosis in CRC, and CD147 blockade disrupts MCT function, elevates intracellular lactate, and suppresses tumor growth, highlighting the MCT–CD147 complex as a therapeutic target (70–72).

#### 2.3.2 Lactate shuttle and tumor–stroma metabolic symbiosis

The lactate shuttle metabolically couples regions with differential oxygenation: hypoxic, glycolytic cells export lactate through MCT4, whereas oxygenated cells import and oxidize it via MCT1 (73, 74). Consequently, glycolytic cells spare glucose for biosynthesis while lactate-consuming cells fuel oxidative phosphorylation, maximizing energy efficiency and tumor growth (74). In CRC, Wnt/β-catenin signaling directly induces MCT1 transcription, linking symbiosis to a canonical driver pathway (75). Moreover, stromal cells contribute to lactate flux: cancer-associated fibroblasts and endothelial cells oxidize tumor-derived lactate and support angiogenesis, while tumor-associated macrophages import lactate and polarize toward immunosuppressive states characterized by Arg1 and IL-10 expression (76–78). Collectively, lactate functions as both metabolic fuel and immunomodulator within the CRC microenvironment (**Figure 2**).

## 3 Lactylation in CRC: beyond histones

### 3.1 Discovery and molecular mechanism

In 2019, Zhang et al. identified a +72 Da modification on histone lysines—Kla—using stable isotope labeling and high-resolution mass spectrometry (15). Kla is induced by elevated intracellular lactate, notably in glycolytically reprogrammed macrophages, and activates wound-healing genes (e.g., Arg1) during inflammatory resolution, establishing lactate as a signaling metabolite and epigenetic regulator (15, 79). Subsequent studies confirmed Kla across species and in disease contexts, including cancer (80, 81). In this subsection, we focus on the donor–writer–eraser–reader framework.

#### 3.1.1 Donor supply for Kla

Enzymatic. L-lactyl-CoA, generated by recently identified lactyl-CoA synthetases, including ACSS2 and nuclear GTPSCS, serves as the donor for enzymatic lactylation; p300 uses L-lactyl-CoA *in vitro*, and p300 knockdown reduces histone Kla in cells (15). Donor availability is further shaped by glycolytic overflow, pyruvate–lactate cycling, and monocarboxylate transport, thereby directly coupling lactate-producing pathways to nuclear acyl-donor pools. In parallel, S-lactoylglutathione (LGSH) from the glyoxalase pathway can non-enzymatically transfer lactyl groups to lysines, particularly under metabolic or oxidative stress, providing a complementary, stress-responsive donor route (82). Together, these routes explain how intracellular lactate becomes an acyl donor capable of encoding metabolic state on chromatin.

#### 3.1.2 Writers (acyltransferases) that install Kla

p300 is the first defined Kla writer, and together with HBO1/KAT7 and related lysine acetyltransferases it installs site-specific Kla in a metabolite-responsive manner (15, 83). These enzymes use lactyl-CoA to transfer the lactyl group to lysine ϵ-amines, elevating transcription-competent chromatin states at loci where lactate-derived donors are abundant. This writer activity provides the forward arm of a dynamic writer–eraser cycle that links donor availability to chromatin activation.

#### 3.1.3 Erasers (delactylases) that remove Kla

Kla is reversible: class I HDACs (HDAC1–3) delactylate histones, and sirtuins (SIRT2, SIRT3) mediate NAD^+^-dependent delactylation with residue selectivity; SIRT3 preferentially targets H4K16la, linking mitochondrial metabolism to nuclear control (84–86). Dynamic opposition between writers and erasers ensures Kla plasticity and allows rapid adaptation to changes in lactate availability. Under sustained glycolytic stress, limited delactylase activity contributes to the persistence of Kla at stress-responsive loci.

#### 3.1.4 Readers that interpret Kla

Bromodomain-containing proteins (e.g., TRIM33) and DPF family members selectively recognize lactylated histones, recruiting chromatin remodelers to activate Kla-enriched promoters and enhancers; H3K18la marks active enhancers in tissue-specific regulation (87–89). Reader engagement connects the chemical mark to transcriptional output, integrating Kla into existing enhancer machinery without necessarily displacing acetyl-lysine signaling. Taken together, donor supply, writers, erasers, and readers constitute a coherent Kla module that links metabolic lactate to chromatin programming. Which substrates and loci are most relevant in CRC, and how they intersect with lactate metabolism, is addressed in the following subsections (Section 3.2).

### 3.2 Evidence of Kla in CRC: histone and non-histone levels

Both histone and non-histone Kla have been identified in CRC, indicating that lactate metabolism governs a dual regulatory system encompassing chromatin remodeling and protein signaling. Representative histone and non-histone lactylation events, together with their residue-level information and evidence levels in CRC, are summarized in **Table 1** and **Supplementary Table 1**. However, it should be noted that while histone lactylation is increasingly supported by CRC patient tissues and functional models, many non-histone lactylation events are still inferred from non-CRC systems and remain to be directly validated in human CRC.

**Table 1:** Evidence levels for key components of the lactate–lactylation axis in CRC.

Axis component/event	Representative molecular node	Primary model/tumour context	Evidence level in CRC
Bevacizumab resistance/angiogenic escape	H3K18la–RUBCNL/VEGFA/ANGPTL4	CRC cell lines; xenografts; anti-VEGF–treated tumours	Confirmed in CRC
Ferroptosis resistance/stemness	H4K12la–GCLC; stemness factors (SOX2, NANOG, AURKB)	CRC stem-like cells; xenografts	Confirmed in CRC
KRAS-driven invasion/metastasis	H3K9la–GRAMD1A and metastasis-associated loci	KRAS-mutant CRC models	Confirmed in CRC
Prognosis/tumour stage	Global H3K18la/H4K12la	CRC tissue cohorts	Confirmed in CRC
TAM-mediated tumour promotion	H3K18la–TRAF6–IL-6–STAT3 axis in TAMs	CRC-associated macrophages (mouse, human)	Confirmed in CRC
Translation and growth	KAT8-dependent lactylation of eEF1A2	CRC cell lines; xenografts	Supported in CRC models
Glycolytic feedback	Lactylation of PFKP and other glycolytic enzymes	Colon cancer cell lines	Supported in CRC models
DNA repair/chemoresistance	MRN-complex lactylation (NBS1, MRE11)	Non-CRC solid tumour models	Extrapolated to CRC
p53 functional modulation	AARS1-mediated p53 lactylation	Multiple non-CRC solid tumours	Extrapolated to CRC
YAP–TEAD oncogenic signaling	YAP lactylation within the Hippo/YAP axis	Hepatocellular and other non-CRC tumours	Extrapolated to CRC
cGAS–STING suppression	cGAS lactylation	Immune and tumour models (non-CRC)	Speculative for CRC
NET-driven inflammation	HMGB1 lactylation	Acute kidney injury and sepsis models	Speculative for CRC
Wound-healing macrophage polarisation	H3K18la-dependent wound-healing programme	Inflammatory macrophage models	Extrapolated to CRC microenvironment

Evidence levels: “Confirmed in CRC” = directly demonstrated in CRC models or patient samples; “Supported in CRC models” = shown in CRC cell lines or xenografts only; “Extrapolated to CRC” = demonstrated in other tumour types and mechanistically inferred for CRC; “Speculative for CRC” = not yet shown in CRC but biologically plausible.

This classification is intended to transparently distinguish evidence strength rather than imply equal levels of validation across tumour types.

Histone lactylation links altered metabolism to gene-expression reprogramming in CRC. Under sustained glycolysis and hypoxia, lactate accumulation enriches H3K18la and H4K12la in CRC tissues, associating with enhanced glycolysis, low-oxygen stress, and therapy resistance (15, 84, 89). Unlike acetylation, which signals nutrient abundance, lactylation is an adaptive chromatin response to metabolic stress (88). Profiling by CUT&Tag and ChIP–seq shows that H3K18la is enriched at transcriptionally active promoters and super-enhancers controlling stress-adaptation programs (76, 80). Clinically, high global histone lactylation correlates with advanced stage, poorer survival, and bevacizumab resistance (16, 33), positioning histone Kla as a tissue-level readout of lactate burden and hypoxic adaptation in CRC.

In parallel, proteome-wide and lactylome studies reveal extensive non-histone lactylation that mirrors lactate flux and oxygen tension. Targets include transcription factors (p53, YAP), metabolic enzymes (HK2, PFKP, PKM2, LDHA), translation factors (eEF1A2), and DNA-repair proteins (NBS1, MRE11), indicating that lactate also rewires cytoplasmic and nuclear signaling pathways (90, 91). Only a subset of these events has been directly validated in CRC models; many were initially described in other tumour types or non-malignant systems and are extrapolated to CRC, as indicated in **Supplementary Table 1**.

Together, histone and non-histone Kla delineate a multilayered regulatory network that couples glycolytic metabolism to gene expression, proteostasis, and therapeutic adaptation in CRC. Accordingly, Section 3.2.1 discusses histone lactylation that governs transcriptional and epigenetic programs, whereas Section 3.2.2 elaborates on non-histone lactylation that coordinates translation, DNA repair, and oncogenic signaling.

#### 3.2.1 Histone Kla in CRC

Lactylation arises from nuclear lactyl-CoA, produced by ACSS2 or the GTPSCS complex, which converts lactate to a CoA-linked donor (92, 93). p300/CBP install lactyl groups when intracellular lactate rises, and HBO1 (KAT7) catalyzes H3K9la/H3K14la in epithelial cancer (93, 94). In CRC, increased expression of p300, HBO1, and ACSS2 parallels H3K18la accumulation in tumour tissues, coupling glycolytic flux to chromatin remodeling and transcriptional activation (95). Removal is mediated by HDAC1–3 and SIRT2/3, which are often downregulated or functionally constrained in hypoxic regions (96–98). TRIM33, BRG1, and DPF2 recognize H3K18la/H3K14la via specialized domains (99). Although most reader studies have been performed in gastrointestinal or pan-cancer models, these mechanisms are likely to extend to CRC and are incorporated into our conceptual framework (**Table 1**; **Supplementary Table 1**). Together, these enzymes and readers form a dynamic circuit that links lactate flux to chromatin accessibility and transcription.

Histone lactylation reprograms CRC transcription to endure metabolic and therapeutic stress by fixing transient metabolic cues into more persistent epigenetic states. Under bevacizumab-induced hypoxia, lactate elevates p300-dependent H3K18la at the RUBCNL promoter, activating autophagy and survival; inhibition of p300 or MCT1/4 reduces H3K18la, suppresses RUBCNL and VEGFA/ANGPTL4, and restores bevacizumab sensitivity (33, 87). In CRC stem-like cells, p300-deposited H4K12la activates GCLC, boosts glutathione, blocks lipid peroxides, and suppresses ferroptosis, linking Kla to redox control (88). More broadly, H3K18la/H4K12la occupy promoters of stemness, EMT, and immune-checkpoint genes, thereby connecting lactate-driven histone Kla to self-renewal, invasion/metastasis, and T-cell evasion in CRC and related gastrointestinal tumours (88, 100–103). Representative loci and functions are summarised in **Supplementary Table 1**. Collectively, histone lactylation establishes a lactate-responsive chromatin landscape that integrates autophagy, ferroptosis resistance, EMT, and immune evasion with therapeutic adaptation.

Prognostically, histone lactylation confers epigenetic plasticity, enabling shifts between proliferative, dormant, and invasive states. Elevated H3K18la/H4K12la associate with advanced stage, lymph-node metastasis, and reduced disease-free survival (16, 93). Co-expression of p300 and H3K18la marks subtypes with poor responses to anti-angiogenic or platinum-based therapy, whereas high SIRT3 or reduced LDHA activity correlates with better outcomes (89, 98). Hence, histone lactylation functions both as a marker and as a mediator of metabolic adaptation in CRC.

#### 3.2.2 Non-histone lactylation in CRC

Non-histone lactylation provides an additional regulatory layer that extends metabolic signaling to proteins controlling translation, DNA-damage responses, oncogenic pathways, and the tumor microenvironment. Lactylomes show widespread modification of transcription factors, metabolic enzymes, translation regulators, and DDR components, with Kla abundance closely tracking lactate flux and oxygen tension (90, 91). The acyl donor lactyl-CoA, synthesized by ACSS2 and the GTPSCS complex, links lactate accumulation to enzymatic modification (93, 94). Multiple acyltransferases—p300/CBP, HBO1/KAT7, KAT8—and AARS1 extend lactylation beyond histones to substrates such as p53 and YAP, while HDAC1–3 and SIRT1/3 mediate delactylation in an NAD^+^- and oxygen-sensitive manner (83, 84, 86, 100, 104, 105). Thus, a dynamic writer–eraser circuit allows lactate to reprogram signaling and proteostasis across compartments. Representative non-histone Kla substrates, their lactylation sites, functional consequences, and evidence levels in CRC are summarised in **Supplementary Table 1**.

In translation control, KAT8-catalysed lactylation of elongation factors such as eEF1A2 enhances protein synthesis and growth under high lactate in CRC models, and is reversible upon KAT8 or MCT1/4 inhibition (105); broader lactylomes implicate additional ribosomal proteins and chaperones, suggesting that lactate adjusts translational capacity to energy supply (90, 91). In DNA-damage responses, lactylation of MRN-complex components including NBS1 and MRE11 has been shown in non-CRC solid tumours to stabilise homologous recombination and increase resistance to genotoxic stress (106); these events are currently extrapolated to CRC (**Supplementary Table 1**). In other solid tumours, AARS1-mediated lactylation of p53 and YAP weakens p53 DNA binding, represses apoptosis genes, and reinforces glycolytic/antioxidant and pro-growth transcriptional programs (104, 107). Lactylation of innate sensors such as cGAS and HMGB1, reported in non-CRC immune and injury models, dampens cytosolic DNA sensing, promotes neutrophil extracellular trap formation, and modulates inflammatory signaling (108–110); these mechanisms provide a plausible link between lactate accumulation and impaired type I interferon signaling in CRC, but remain to be directly validated (**Supplementary Table 1**).

In the tumor microenvironment, macrophage H3K18 lactylation silences RARγ and activates the TRAF6–IL-6–STAT3 cascade, promoting M2 polarization and immunosuppression, particularly in microsatellite-stable CRC (43, 111). At the metabolic level, CRC lactylomes identify Kla on HK2, PFKP, PKM2, and LDHA, evidencing reciprocal control between glycolysis and lactate flux; PFKP lactylation appears to reduce catalytic activity, providing negative feedback that prevents overload while preserving lactate pools for signaling (90, 91). Moreover, MCT4-dependent lactate export from fibroblasts and MCT1-mediated uptake in tumor cells establish lactate-rich niches where coordinated histone and non-histone lactylation drive stromal communication, epithelial plasticity, and drug resistance (45, 112–114). Taken together, non-histone lactylation integrates metabolism with translation, DNA repair, oncogenic transcription, and immune evasion, complementing chromatin-based regulation.

### 3.3 Crosstalk with other epigenetic marks

Lactylation operates within an integrated epigenetic network, mirroring lactate flux and oxygen availability and thereby reflecting tumor metabolic state (15, 81). Because Kla is written and erased by enzymes such as p300/CBP, HBO1/KAT7 and HDAC1–3, which also regulate acetylation and other short-chain acylations, it is intrinsically coupled to broader chromatin remodeling under sustained glycolysis and hypoxia (83, 84, 94, 100).

At the cofactor level, lactyl-CoA generated during glycolytic overflow competes with acetyl-CoA and other acyl donors for lysine modification on histone and non-histone substrates (94, 115). Lactylome studies in gastrointestinal tumours identify thousands of Kla sites whose abundance tracks lactate levels and oxygen tension, underscoring metabolic control of acylation stoichiometry (81). Shared writers (p300/CBP, HBO1/KAT7) and delactylases (HDAC1–3, SIRT1/3) also install or remove acetyl and other acyl marks (83, 84, 100, 116–118), creating competition and cooperation at common lysines. Spatial profiling shows that H3K18la frequently co-localises with H3K27ac and H3K4me3 at active promoters and super-enhancers, illustrating crosstalk between lactylation and activating chromatin configurations (81, 89). Beyond acetylation, one-carbon metabolism, DNA and RNA methylation, and other short-chain acylations (such as crotonylation and propionylation) share metabolic inputs with Kla, while microbiota-derived short-chain fatty acids can favour alternative acylation states in colonic epithelium (116, 119, 120). Collectively, these layers position lactylation as a flexible node within an acylation–methylation network that links carbon flux to epigenomic plasticity in CRC (**Table 1**, **Supplementary Table 1**).

## 4 Functional integration of the Lactate–Lactylation axis in CRC progression

### 4.1 EMT, invasion and metastasis

#### 4.1.1 Histone lactylation primes EMT transcriptional programs

In CRC, hypoxia-driven glycolysis elevates lactate and nuclear lactyl-CoA, enriching H3K18la and H4K12la at promoters and enhancers of EMT transcription factors and motility genes (e.g., SNAI1, ZEB1), with Kla levels tracking EMT scores and invasive fronts across models and patient cohorts (15, 81). As described in Section 3, H3K18la acts as a stress-responsive layer that partly overlaps but is kinetically distinct from H3K27ac, and limited delactylation by HDAC1–3 and sirtuins under hypoxia permits persistence of these marks (15, 84, 121). p300/CBP and HBO1/KAT7 install EMT-linked histone Kla, while TRIM33- and DPF2-containing complexes read Kla within SWI/SNF modules to shape enhancer accessibility (83, 87, 88). Clinically, higher global Kla and H3K18la/H4K12la expression associate with advanced stage, nodal involvement, and shorter disease-free survival, consistent with a role in sustaining invasiveness in CRC (81, 107).

#### 4.1.2 Non-histone lactylation augments motility and proteostasis

In other solid tumours, YAP lactylation increases nuclear retention and TEAD-dependent transcription, providing a plausible mechanism by which lactate could reinforce pro-migratory programs in CRC (**Supplementary Table 1**) (105, 122). Non-histone Kla links metabolic state to signaling strength and protein output. In CRC models, KAT8-dependent lactylation of elongation factors such as eEF1A2 enhances translational elongation and supports cell migration and tumour growth; inhibition of KAT8 or MCT1/4 reduces these motility phenotypes (91). Lactylomes also identify Kla on glycolytic enzymes (PFKP, PKM2, LDHA) and chaperones, indicating that glycolytic flux drives Kla, which in turn modulates proteostasis and redox/ATP balance during migration and matrix remodelling (114, 123). At the tumour–stroma interface, MCT1/MCT4-mediated lactate shuttling between CRC cells and CAFs amplifies the lactate–lactylation axis, and spatial/single-cell analyses consistently map EMT-high regions to lactate-responsive transcripts and activated fibroblast states at invasion fronts (13, 30, 124–127). Key non-histone EMT-related substrates and their evidence levels in CRC are summarised in **Supplementary Table 1**.

#### 4.1.3 Metastatic seeding and organ microenvironments (liver-prone niche)

Export and conditioning: At invasive fronts, MCT4-driven lactate export correlates with stromal remodeling and dissemination potential (128). In the liver, tumour-derived lactate activates hepatic stellate cells, increases collagen deposition and alters sinusoidal architecture, thereby lowering the threshold for CRC cell colonisation and metastatic outgrowth (83, 129). Lactate also contributes to a pre-metastatic immune niche by promoting M2-like macrophage programs and impairing NK-cell cytotoxicity, aligning metabolic stress with early immune evasion (124, 130, 131). Multi-region and spatial omics studies link lactate-high zones with EMT-high tumour cells and activated stroma in primary CRC and liver lesions, supporting a model in which the lactate–lactylation axis coordinates local invasion with organ-specific niche conditioning (126, 132–134). Emerging metabolic imaging approaches, such as hyperpolarized [1-^13C]pyruvate MRI, can non-invasively quantify glycolytic flux *in vivo* and may enable monitoring of lactate-driven programs during invasion and dissemination in CRC (135, 136).

### 4.2 Angiogenesis and therapy resistance

#### 4.2.1 Lactate–lactylation rewires angiogenic programs

Hypoxia and anti-angiogenic pressure intensify glycolysis in CRC, elevating lactate and the nuclear lactyl-donor pool for histone lactylation (Kla). In endothelial cells, VEGF rapidly raises H3K9la and—together with HDAC2—creates a feed-forward loop that amplifies tip-cell programs and sprouting; inhibiting glycolysis or lactate transport suppresses H3K9la and neovascularization (137). In tumor cells, H3K18la/H4K12la accumulates at VEGFA/KDR/ANGPT enhancers, adding a metabolite-encoded activation layer on top of H3K27ac (15). Kla at angiogenic loci is installed by p300/CBP and HBO1/KAT7 and interpreted by TRIM33- and DPF2-containing complexes that integrate into BRD4-based super-enhancer scaffolds without dismantling them (83, 87, 88, 138, 139). Parallel signaling reinforces these chromatin effects: lactate stabilizes HIF outputs and signals via GPR81, boosting VEGF and tip-cell markers (DLL4, ESM1) in ECs and cancer cells (140, 141). Spatially, MCT1/MCT4-mediated tumor–stroma shuttling enriches lactate-responsive angiogenesis signatures at invasive fronts, and spatial omics localize lactate-high zones to activated endothelium and CAF niches in CRC and liver lesions (45, 142–144).

#### 4.2.2 Autophagy coupling drives adaptive resistance to anti-VEGF

Bevacizumab reduces perfusion yet deepens hypoxia, sustaining lactate and Kla. Mechanistically, lactate-induced H3K18la upregulates the autophagy enhancer RUBCNL, increases LC3/ATG flux, and preserves tumor/EC viability during VEGF blockade—facilitating vascular rebound and progression (16). Accordingly, constraining lactate flux (LDHA or MCT1/4 inhibition) or dampening writer activity reduces H3K18la at autophagy/angiogenesis loci and delays re-vascularization (16, 45, 145). Under hypoxia, reduced HDAC1–3-mediated delactylation allows Kla to outlast acetylation, providing an epigenetic “memory” that hastens reactivation of angiogenic programs between treatment cycles (84, 137). In parallel, non-histone Kla supports resistance: KAT8-dependent lactylation of eEF1A2 enhances translation of pro-angiogenic proteins and matrix components required for sprouting and lumen maturation under stress (105). Together, chromatin remodelling, proteostasis and lactate transport sustain angiogenesis despite VEGF receptor blockade, providing a rationale for combining anti-angiogenic agents with LDHA, MCT1/4 or p300/CBP inhibitors that target the lactate–lactylation axis.

### 4.3 Immunosuppression and immune escape

**Figure 3:**
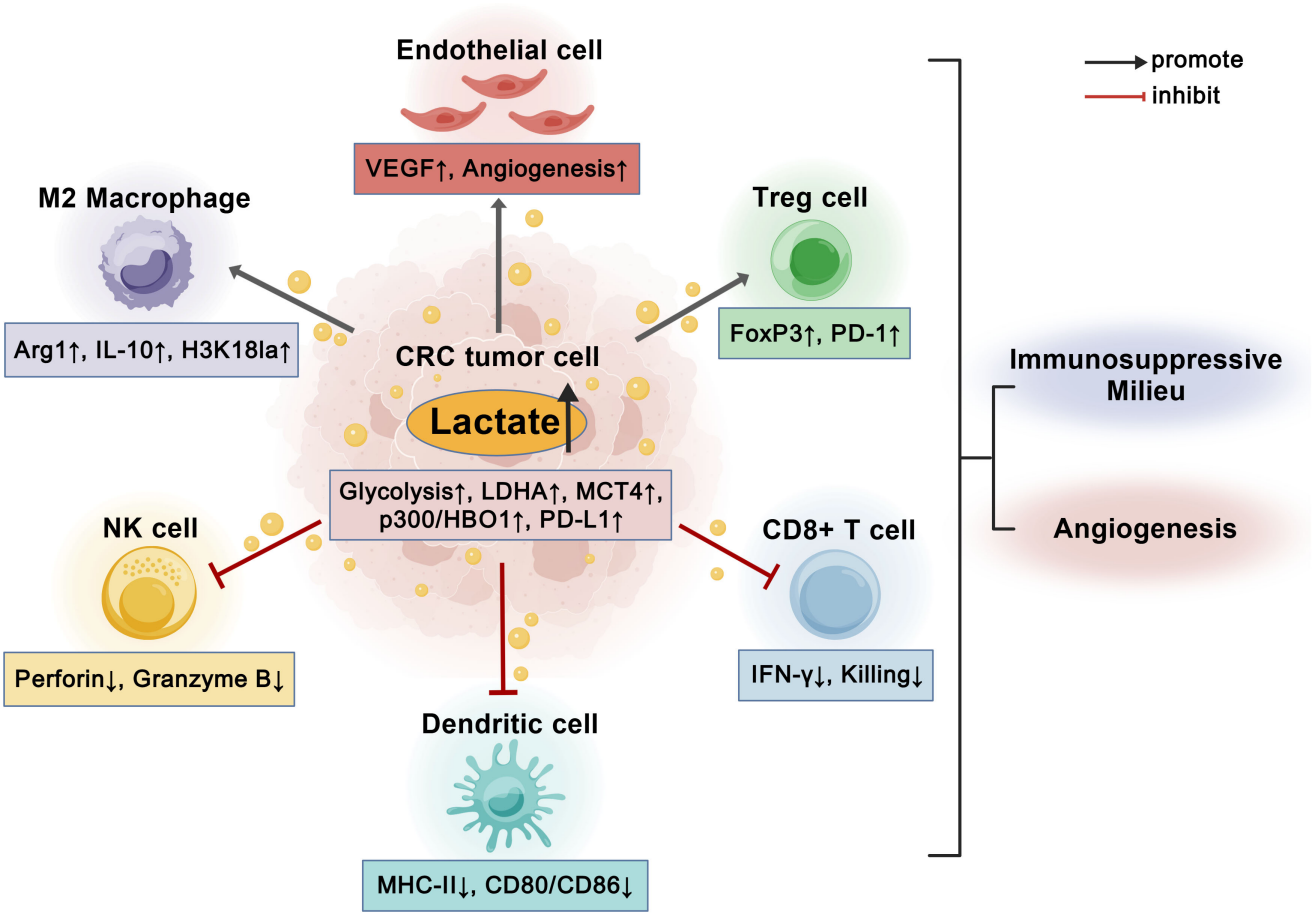
The lactate–lactylation axis orchestrates immune suppression and angiogenesis in CRC. Excess lactate produced by glycolytic CRC cells (via LDHA, MCT4, and p300/HBO1 activation) accumulates in the tumor microenvironment and acts as a central mediator linking metabolism to immune modulation. Lactate and histone lactylation (H3K18la) in tumor and stromal cells induce M2 macrophage polarization (Arg1↑, IL-10↑), enhance Treg activity (FoxP3↑, PD-1↑), and impair cytotoxic immune responses by suppressing CD8^+^ T cells (IFN-γ↓, killing↓), NK cells (Perforin↓, Granzyme B↓), and dendritic cell activation (MHC-II↓, CD80/CD86↓). Concurrently, lactate-driven lactylation upregulates VEGF in endothelial cells, promoting angiogenesis. Collectively, these processes establish an immunosuppressive and pro-angiogenic milieu that facilitates CRC progression. Created with BioGDP.com. CRC, colorectal cancer; LDHA, lactate dehydrogenase A; MCT, monocarboxylate transporter; HBO1, histone acetyltransferase binding to ORC1 (KAT7); H3K18la, histone H3 lysine-18 lactylation; Arg1, arginase-1; IL-10, interleukin-10; FoxP3, forkhead box P3; PD-1, programmed cell death protein 1; IFN-γ, interferon-gamma; NK, natural killer; MHC-II, major histocompatibility complex class II; CD80/CD86, costimulatory molecules; VEGF, vascular endothelial growth factor.

In CRC, hypoxia and anti-vascular stress raise intratumoral lactate, expanding the acyl-donor pool for histone Kla in myeloid cells. In tumour-associated macrophages, H3K18la accumulates with delayed kinetics distinct from acetylation and reprograms transcription toward wound-healing/tolerogenic modules (e.g., ARG1), sustaining M2-like polarisation; hypoxia-constrained HDAC1–3 activity prolongs these immunosuppressive states (15, 84) (**Figure 3**). Increased protein lactylation also dampens cytosolic DNA sensing by curtailing cGAS activity, thereby lowering cGAMP/type-I IFN production, dendritic-cell priming and chemokine recruitment (108). Natural killer (NK) cells are acutely sensitive to lactate and acidosis: elevated extracellular lactate impairs glycolysis and mitochondrial function and suppresses granzyme/perforin-dependent cytotoxicity, undermining innate effector responses in the TME (13, 26, 30). Clinical and preclinical studies implicate lactate accumulation and Kla as key barriers to NK/CTL effector function and contributors to immunotherapy failure. Spatially, MCT1/MCT4-mediated lactate shuttling at invasive fronts and perivascular niches coincides with NK exclusion, VEGF-high/M2-rich microdomains and weak chemokine gradients, linking angiogenesis and myeloid tolerance along a single metabolic axis (124, 126, 146).

T-cell control is similarly constrained. Lactate-rich, glycolysis-high tumours favour Treg differentiation and stability while suppressing CD8^+^ T-cell cytolysis and cytokine production (87, 147, 148) (**Figure 3**). Tregs in these niches further upregulate PD-1, reinforcing checkpoint inhibition (45). On the tumour side, lactate–GPR81 signaling and inflammatory circuits—particularly IL-6–JAK–STAT3—converge on PD-L1 upregulation; CRC data show strong STAT3-driven PD-L1 transcription and immune evasion (125, 149). Spatial transcriptomic and metabolomic maps align lactate-high regions with Treg enrichment, M2-TAM markers, diminished type-I IFN transcripts and elevated PD-L1—features typical of pMMR/MSS CRC and predictive of poor PD-1/PD-L1 responses (126, 150, 151). These observations suggest testable strategies: reduce lactate flux (MCT1/4, LDHA), accelerate Kla turnover (HDAC1–3), or block GPR81/STAT3 signaling to restore innate sensing, destabilize Tregs, and sensitize MSS CRC to immunotherapy.

## 5 Therapeutic strategies targeting the lactate–lactylation axis

### 5.1 Targeting lactate production and transport

Disrupting the lactate axis in CRC requires dual targeting of supply (pyruvate→lactate) and shuttling (MCT-mediated transport) to break stromal–tumor symbiosis, relieve immunosuppression and enhance responses to targeted therapies and immunotherapy, particularly in MSS CRC. Notably, prior clinical experience indicates that single-enzyme metabolic inhibitors have generally shown limited efficacy when used in isolation, underscoring the need for multi-target and combination-based approaches (152). Representative metabolic and transport targets along this axis are summarised in **Table 2**.

**Table 2:** Therapeutically actionable nodes along the lactate–lactylation axis in CRC.

Category	Target	Mechanistic role	Modality	Development status	References
I. Lactate Production	LDHA	Pyruvate→lactate; sustains glycolysis; favors immune evasion and angiogenesis	LDHA inhibitors (GNE-140; monoamide chemotypes)	Preclinical	(21, 153–156)
	PDK1/2	PDH inactivation; carbon diverted to lactate	DCA; PDK modulators	Preclinical; 5-FU resensitization	(30–33, 157)
	PKM2	Increases glycolytic flux; nuclear co-activation of β-catenin and MYC	TEPP-46	Preclinical	(24, 25, 97, 158)
	PCK1/2	Prevents lactate utilization & tumor growth	PCK inhibitors	Concept	(15)
	GLUT1	Elevated glucose influx → lactate burden	GLUT1 inhibitors	Preclinical association	(28)
	PFKFB3	Boosts glycolysis & angiogenesis	PFKFB3 inhibitors	Preclinical	(29)
II. Lactate Transport and Signaling	MCT1	Lactate import; tumor–stroma symbiosis	AZD3965; AR-C155858	Phase I	(96, 158–160)
	MCT4	Lactate/proton export; acidification	Dual MCT1–MCT4 ± metformin	Preclinical	(37, 97, 161)
	CD147	Stabilizes MCT1–MCT4	Anti-CD147 antibody	Preclinical	(47, 162)
	GPR81/HCAR1	Lactate receptor → PD-L1 & VEGF	HCAR1 antagonists	Concept	(26, 146)
	LDHB	Lactate→pyruvate; biomarker for anti-EGFR	Biomarker development	Translational biomarker	(26)
III. Lactyl-CoA Formation and Lactylation Machinery	ACSS2	Generates lactyl-CoA; fuels histone lactylation	ACSS2 inhibitors/degraders	Preclinical	(67, 101)
	GTPSCS	Nuclear lactyl-CoA synthetase	Probe development	Preclinical	(66)
	EP300/CBP	Histone Kla writers	HAT inhibitors; p300 degraders	Early clinical; preclinical CRC	(57, 59, 66, 67)
	HBO1/KAT7	Catalyzes H3K9/14 lactylation	KAT7 inhibitors	Preclinical	(61)
	KAT8/MOF	Lactylates eEF1A2 → ↑ protein synthesis	KAT8 inhibitors	Preclinical	(87)
	AARS1	Lactylates TP53/YAP	AARS1 inhibitors	Preclinical	(86)
	HDAC1-3	Delactylases; regulate Kla turnover	HDAC modulators	Concept	(58, 89)
	SIRT1/3	NAD-dependent delactylation	Sirtuin activators	Preclinical	(73, 88, 107)
	TRIM33	Reads histone lactylation	Reader blockade	Preclinical	(62)
	DPF2	Reads H3K14la; tumorigenesis	Reader degradation	Preclinical	(60)
	BRG1	Chromatin remodeling with Kla	Reader/chromatin inhibitors	Preclinical	(62)
IV. Downstream Effectors Modified by Lactylation	NBS1/MRE11	Kla stabilizes MRN → HR repair; chemoresistance	Reduce lactylation; PARP/platinum	Preclinical	(16)
	YAP	K90la → TEAD activation	TEAD inhibitors; AARS1 targeting	Preclinical	(87, 90),
	TP53	K382la weakens DNA binding	SIRT1 activation; AARS1 inhibition	Preclinical	(73, 86)
	cGAS	Kla suppresses type I IFN	STING agonists	Preclinical	(91)
	HMGB1	Kla promotes NET formation	Delactylation; lactate lowering	Preclinical	(92, 93)
	ULK2	Supports migration via MCT4	ULK inhibitors	Preclinical	(95)
	ANTXR1	Lactylation → oxaliplatin resistance	ANTXR1 inhibition	Preclinical	(193)
	BRD4	Maintains SE output; cooperates with Kla	BET inhibitors	Early clinical; synergy	(84, 86, 104, 105, 136, 137)

This table summarizes key metabolic, transport, and chromatin-associated regulatory nodes that constitute the lactate–lactylation axis in CRC. Targets are organized into four functional categories: (I) lactate production; (II) lactate transport and extracellular signaling; (III) lactyl-CoA generation and the enzymatic machinery controlling histone and non-histone lactylation; and (IV) downstream effectors whose activities are directly modulated by lactylation. For each target, representative mechanistic roles in CRC, corresponding therapeutic modalities, and current stages of translational development (clinical trial, preclinical, or conceptual) are provided. This categorization highlights both clinically advanced interventions (e.g., MCT1 inhibition with AZD3965) and emerging epigenetic nodes (e.g., EP300/CBP, AARS1, TRIM33, DPF2) within the lactate–lactylation regulatory network, most of which remain at a preclinical or early clinical stage and require careful evaluation of therapeutic windows and long-term safety..

ACSS2, acyl-CoA synthetase short-chain family member 2; AARS1, alanyl-tRNA synthetase 1; BET, bromodomain and extra-terminal motif; BRD4, bromodomain-containing protein 4; CBP, CREB-binding protein; cGAS, cyclic GMP-AMP synthase; DDR, DNA damage response; DPF2, double PHD fingers 2; eEF1A2, eukaryotic elongation factor 1A2; EGFR, epidermal growth factor receptor; EP300, E1A-binding protein p300; GLUT1, glucose transporter 1; GTPSCS, GTP-succinyl-CoA synthetase complex; HAT, histone acetyltransferase; HCAR1, hydroxycarboxylic acid receptor 1; HDAC, histone deacetylase; HMGB1, high-mobility group box 1; ICB, immune checkpoint blockade; Kla, lysine lactylation; LDH, lactate dehydrogenase; LDHA, lactate dehydrogenase A; LDHB, lactate dehydrogenase B; MCT, monocarboxylate transporter; METTL3, methyltransferase-like 3; MRN, MRE11-RAD50-NBS1 complex; NAD, nicotinamide adenine dinucleotide; PARP, poly ADP-ribose polymerase; PDH, pyruvate dehydrogenase; PDK, pyruvate dehydrogenase kinase; PFKFB3, 6-phosphofructo-2-kinase/fructose-2,6-bisphosphatase 3; PCK, phosphoenolpyruvate carboxykinase; PKM2, pyruvate kinase M2; SE, super-enhancer; SIRT, sirtuin; STAT3, signal transducer and activator of transcription 3; TEAD, TEA domain transcription factor; TP53, tumor protein p53; ULK2, unc-51-like kinase 2; VEGF, vascular endothelial growth factor; YAP, Yes-associated protein.

#### 5.1.1 Suppressing lactate supply

Pharmacologic suppression of lactate production in CRC focuses mainly on LDH, PDK and PKM2 (**Table 2**). LDH inhibition (e.g., with tool compounds such as GNE-140) limits lactate generation, reduces tumour glucose uptake, increases glucose availability for effector T cells and potentiates checkpoint blockade, thereby reversing the high-lactate/low-glucose gradient that suppresses immunity (153). Structure-guided LDHA inhibitors with drug-like properties are in preclinical development and, in models, can also restore dendritic, NK and CTL function, supporting their incorporation into regimens for immune-refractory CRC (154–156).

Metabolic re-routing complements LDH blockade. PDK inhibition with dichloroacetate (DCA) activates pyruvate dehydrogenase, channels pyruvate into mitochondrial oxidation, dampens aerobic glycolysis and can resensitise CRC cells to fluorouracil in preclinical models (157). PKM2 activators such as TEPP-46 stabilise the tetrameric enzyme, constrain its non-metabolic nuclear functions and modulate myeloid and tumour immunometabolism (45, 158). Together, LDH, PDK and PKM2 targeting reduces lactate burden, partially restores oxidative balance and may enhance anti-tumour immunity in CRC.

#### 5.1.2 Interrupting lactate shuttling

MCT1 and MCT4 coordinate proton-coupled lactate flux between glycolytic exporters and oxidative importers and are associated with aggressive disease and poor outcomes in CRC (45, 145, 159) (**Table 2**). The MCT1 inhibitor AZD3965 has entered clinical testing: first-in-human studies demonstrated target engagement and a tolerable exposure window, although on-target expression of MCT1 in retina and myocardium constrains dosing (158, 160).

Preclinical CRC models further indicate that cetuximab-resistant tumors become dependent on MCT1-mediated lactate recycling; MCT1 blockade (e.g., with AR-C155858) suppresses uptake/oxidation and growth, nominating MCT1 as a druggable vulnerability in anti-EGFR-refractory disease (113). Combinations of dual MCT1/4 inhibition (e.g., syrosingopine) with complex I inhibitors such as metformin collapse redox homeostasis and induce synthetic lethality in glycolysis-addicted settings (161). Additional strategies, including targeting the MCT chaperone basigin (CD147) with antibodies to disrupt MCT trafficking and ultrasound-responsive microbubbles to enhance intratumoral delivery of AZD3965, remain at the preclinical proof-of-concept stage (162, 163).

#### 5.1.3 Biomarker-guided application and combination therapy design

Baseline serum LDH, a negative prognostic marker in large metastatic CRC cohorts, together with tumour expression of MCT1/4 and CD147 and functional imaging of pyruvate→lactate flux by hyperpolarized [1-¹³C]-pyruvate MRI, can help identify patients with a high lactate burden who are most likely to benefit from lactate-axis interventions and provide early pharmacodynamic readouts (47, 164, 165).

Because physiological MCT1 in retina and myocardium limits systemic exposure to transport inhibitors, rational sequencing that uses LDH/PDK “metabolic priming” followed by MCT1 ± MCT4 blockade may mitigate ocular and cardiac risk while preserving tumour selectivity (159, 160, 163). Overall, a dual-axis strategy that combines lactate production blockade (LDH, PDK, PKM2) with transport inhibition (MCT1/4 with or without anti-CD147) offers a feasible approach to disrupt tumour–stroma symbiosis, relieve lactate-driven immunosuppression and resensitise CRC—particularly MSS disease—to anti-VEGF, anti-EGFR and immune checkpoint therapies, as outlined in **Table 2**.

### 5.2 Targeting the lactylation machinery

Kla couples glycolytic overflow to durable gene activation in gastrointestinal cancers, mapping to active promoters/enhancers (e.g., H3K18la) and linking metabolic stress to therapy resistance (16, 33, 89). This positions donor supply, writers, erasers and readers as druggable nodes in CRC, with representative interventions along this module summarised in **Table 2**.

#### 5.2.1 Donor supply and writers: preventing Kla installation

Kla depends on nuclear lactyl-CoA generated by ACSS2 and the GTPSCS complex, which fuel histone and non-histone lactylation and can cooperate with KAT2A to promote immune evasion (93, 94). Inhibiting or degrading ACSS2/GTPSCS is therefore a plausible way to deplete the lactyl donor pool, although current approaches remain preclinical. Among writers, p300/CBP are the best-characterised enzymes installing histone Kla, and small-molecule p300/CBP inhibitors already in early-phase oncology trials could, in principle, be repurposed to attenuate Kla in CRC (83, 84). HBO1/KAT7 and KAT8/MOF extend this writer repertoire to additional histone and non-histone substrates, including translational factors such as eEF1A2 in high-lactate settings (105). Beyond KATs, AARS1 has been identified as a lactate-sensitive lactyltransferase for p53 and YAP in other tumour models, weakening p53 DNA binding and reinforcing pro-growth signaling, but remains a conceptual target without CRC-specific inhibitors (104). In CRC, writer-dependent circuits—in which H3K18la drives RUBCNL and bevacizumab resistance, H4K12la activates GCLC to suppress ferroptosis and maintain stemness, and KRAS-driven H3K9la at GRAMD1A promotes metastasis—link these enzymes to clinically relevant phenotypes (Sections 3–4 and **Supplementary Table 1**) (16, 33, 92).

#### 5.2.2 Erasers and turnover engineering: accelerating delactylation

Kla is reversible. Class I HDACs (HDAC1–3) and sirtuins (SIRT1/SIRT3) delactylate with residue selectivity; SIRT3 targets H4K16la and links mitochondrial redox to nuclear acyl turnover, while SIRT1/SIRT3 regulate histone and non-histone lactylation (86, 100). Under hypoxia, however, delactylase activity is constrained and stress-induced Kla can accumulate as an epigenetic “memory” at responsive loci. This motivates “turnover engineering”: the development of deacylase modulators that preferentially enhance delactylation while sparing essential acetylation, although such agents are currently limited to preclinical tools.

#### 5.2.3 Readers and chromatin interpretation: disabling Kla-dependent enhancer programs

Kla acts via selective recognition. Validated readers include the TRIM33 bromodomain (histone Kla) and DPF2 (PHD–BRD module recognizing H3K14la) that recruit remodelers to activate transcription (87, 88). Multi-omic maps show that Kla often co-localizes with H3K27ac/H3K4me3 at super-enhancers but displays distinct kinetics, forming a lactate-responsive activation layer rather than a simple acetylation surrogate (89, 98). Non-histone Kla on factors such as NBS1, YAP, cGAS and HMGB1 further links lactylation to DNA repair, TEAD signaling and innate immune responses, although much of this evidence derives from non-CRC models (**Supplementary Table 1**) (106–108, 166). Collectively, these findings identify TRIM33/DPF2-containing complexes as potential reader targets that could complement donor- and writer-focused interventions, though reader-focused drug development is still at a conceptual stage.

### 5.3 Combination and translational opportunities

#### 5.3.1 Backbone therapies plus lactate–lactylation control

Anti-angiogenic therapy creates hypoxic, glycolytic niches that elevate lactate and histone lactylation, activating autophagy and drug-tolerance programs. In CRC, bevacizumab induces H3K18la-driven RUBCNL transcription, sustaining autophagy and resistance, which supports combining anti-VEGF with agents that lower lactate production (LDH/PDK/PKM2 inhibitors) or block Kla installation (p300 and related writers) (16, 113, 167). Likewise, cetuximab resistance generates MCT1-dependent lactate recycling, and pharmacologic MCT1 inhibition suppresses lactate uptake/oxidation and tumour growth, nominating MCT1 as a vulnerability in anti-EGFR–refractory disease (113). Metabolically, LDH inhibition diverts glucose back to effector T cells and improves checkpoint blockade, whereas PDK inhibition restores pyruvate oxidation and enhances fluoropyrimidine efficacy, together supporting short-course “metabolic priming” with LDH/PDK modulators before or alongside standard backbones (16, 153, 168). Dual MCT1/4 blockade combined with complex-I inhibition (e.g., metformin) collapses redox homeostasis in glycolysis-addicted models, and early AZD3965 studies show on-target pharmacodynamics with a manageable safety window, supporting staged combinations that respect physiological MCT1 expression in retina and myocardium (161, 169).

#### 5.3.2 Microenvironment, delivery and microbiome

CRC features CAF–tumor lactate shuttles that promote invasion, angiogenesis, oxaliplatin resistance, and T-cell exclusion. Disrupting these circuits with MCT1/4 inhibition (such as AZD3965 in preclinical models) reduces motility and pro-angiogenic signaling in co-culture and *in vivo* (170, 171). The intestinal microbiome can bias luminal and portal lactate flux and remodel mucosal immunity; dietary or probiotic strategies that lower net lactate are being explored as low-intensity adjuncts to pharmacological lactate-axis control (163, 172). To widen the therapeutic window for transport inhibitors, ultrasound-responsive microbubbles can deliver AZD3965 focally and increase intratumoural exposure, while basigin (CD147) antibodies convert the MCT chaperone into a negative modulator, suppressing transport and enhancing antitumour immunity in combination with small-molecule MCT1/4 inhibitors (162, 165). Together, these approaches support metabolism-aware regimens that can be tuned for efficacy and safety across heterogeneous lesions, including liver metastases.

#### 5.3.3 Biomarkers and trial design

A biomarker backbone should integrate serum LDH (prognostic; metabolic load), IHC for H3K18la/H4K12la, and expression of MCT1/4–CD147 and lactylation writers/donors (p300/HBO1/ACSS2/GTPSCS) to stratify patients for lactate-axis combinations (16, 113, 156). Hyperpolarized [1-^13C]-pyruvate MRI quantifies pyruvate→lactate flux within minutes, and emerging multicenter frameworks support its use as a sensitive pharmacodynamic readout for LDH/MCT inhibition and as a bridge to tissue lactylation endpoints (133, 173). We propose a pragmatic treatment sequence of metabolic priming → transport blockade → epigenetic consolidation: short-course LDH/PDK priming lowers lactate supply, improves T-cell fuel access and increases fluoropyrimidine sensitivity (e.g., LDH inhibition boosts ICB, DCA resensitizes CRC to 5-FU) (16, 153); subsequent MCT1 ± MCT4 blockade (± anti-CD147) interrupts tumour–stroma shuttling; and, where indicated, writer/reader/BET targeting extinguishes lactate-imprinted programs (87, 88, 160, 162). Window-of-opportunity trials that embed HP-^13C MRI with on-treatment biopsies (Kla IHC and, in selected cohorts, ChIP-seq or lactylomes) can align flux changes with epigenetic remodelling and refine scheduling. Within this biomarker-guided framework, lactate–lactylation–directed combinations have the potential to resensitise anti-EGFR–refractory disease and potentiate immune checkpoint blockade in CRC (113, 153) (**Table 2**).

## 6 Conclusions and future prospects

Lactate and Kla are central to CRC biology. They couple glycolytic overflow to chromatin remodeling and immune escape, shaping a tumor microenvironment that is acidic, angiogenic, and therapy resistant. Across this review, we outline a practical framework that targets the lactate–lactylation–immunity axis at three levels: limiting lactate production (LDH/PDK/PKM2), blocking lactate shuttling (MCT1/4 with or without anti-CD147), and interrupting Kla signaling (donor/writer inhibition, delactylase activation, reader blockade). These interventions are complementary. Together they aim to dismantle metabolic symbiosis, reset oncogenic transcription, and restore antitumor immunity.

Clinical translation should be biomarker-guided. Serum LDH reflects metabolic load. Tissue markers such as H3K18la/H4K12la, and expression of MCT1/4–CD147 and writers/donors (p300/HBO1/ACSS2/GTPSCS), can stratify patients. Hyperpolarized [1-^13C]-pyruvate MRI provides a rapid, noninvasive readout of pyruvate→lactate flux and can be paired with on-treatment biopsies to align metabolic responses with Kla dynamics. Safety requires attention to on-target transporter expression in the retina and myocardium and to systemic effects of glycolysis modulation.

Future work should focus on three goals. First, define exposure–response relationships and optimal sequencing in window-of-opportunity trials using integrated imaging and tissue pharmacodynamics. Second, delineate writer/eraser/reader dependencies across consensus molecular subtypes and liver metastases using spatial multi-omics, and validate causal, *in vivo* roles of the lactate–lactylation axis in genetically engineered mouse models. Third, advance small molecules and biologics with improved selectivity and eye- and brain-sparing profiles. Integrating lactate metabolism and lactylation into CRC taxonomies may open new therapeutic windows—particularly for microsatellite-stable and metastatic disease—and convert mechanistic insight into durable clinical benefit.

## Glossary

AARS1: Alanyl-tRNA synthetase 1
ACSS2: Acyl-CoA synthetase short-chain family member 2
ANTXR1: Anthrax toxin receptor 1
ATP: Adenosine triphosphate
BET: Bromodomain and extra-terminal motif
BRD4: Bromodomain-containing protein 4
CBP: CREB-binding protein
cGAS: Cyclic GMP–AMP synthase
CRC: Colorectal cancer
CSC: Cancer stem cell
DCA: Dichloroacetate
DDR: DNA damage response
DPF2: Double PHD fingers 2
eEF1A2: Eukaryotic elongation factor 1A2
EGFR: Epidermal growth factor receptor
EMT: Epithelial–mesenchymal transition
EP300: E1A-binding protein p300
GLUT1: Glucose transporter 1
GTPSCS: GTP-succinyl-CoA synthetase complex
H3K9: H3K14, H4K16, Specific lysine residues on histone H3 or H4 (sites of lactylation or acetylation)
HAT: Histone acetyltransferase
HCAR1: Hydroxycarboxylic acid receptor 1 (GPR81)
HDAC: Histone deacetylase
HMGB1: High-mobility group box 1
ICB: Immune checkpoint blockade
KAT2A: KAT7, KAT8, Lysine acetyltransferase family members (also known as GCN5, HBO1, MOF)
Kla: Lysine lactylation
LDH: Lactate dehydrogenase
LDHA: Lactate dehydrogenase A
LDHB: Lactate dehydrogenase B
METTL3: Methyltransferase-like 3
MCT: Monocarboxylate transporter
MCT1 (SLC16A1): Monocarboxylate transporter 1
MCT4 (SLC16A3): Monocarboxylate transporter 4
MOF (KAT8): Males absent on the first, lysine acetyltransferase 8
MRN: MRE11–RAD50–NBS1 complex
MYC: Myelocytomatosis oncogene
NAD: Nicotinamide adenine dinucleotide
NBS1: Nijmegen breakage syndrome 1
PARP: Poly(ADP-ribose) polymerase
PDH: Pyruvate dehydrogenase
PDK1/2: Pyruvate dehydrogenase kinase 1/2
PFKFB3: 6-Phosphofructo-2-kinase/fructose-2,6-bisphosphatase 3
PCK1/2: Phosphoenolpyruvate carboxykinase 1/2
PKM2: Pyruvate kinase M2
ROS: Reactive oxygen species
SIRT1/3: Sirtuin 1/3 (NAD-dependent deacetylases and delactylases)
STAT3: Signal transducer and activator of transcription 3
STING: Stimulator of interferon genes
TCA: Tricarboxylic acid cycle
TEAD: TEA domain transcription factor
TP53 (p53): Tumor protein p53
TRIM33: Tripartite motif-containing 33
ULK2: Unc-51-like kinase 2
VEGF: Vascular endothelial growth factor
YAP: Yes-associated protein
