# Supplementary material for: Lactate metabolism and protein lactylation in colorectal cancer: from metabolic reprogramming to epigenetic regulation
Source: Front Oncol. 2026 Mar 3;16:1741782. doi: 10.3389/fonc.2026.1741782 (PMC12993827; doi:10.3389/fonc.2026.1741782)
Supplement: Supplementary file 1 [file Table1.docx]

**Supplementary Table S1 Representative histone and non-histone lactylation events relevant to CRC**

| Class | Substrate / histone mark | Residue(s) / locus | | Functional consequence | Primary model / tumour context | Evidence in CRC | References |
| --- | --- | --- | --- | --- | --- | --- | --- |
| Histone | H3K18la–RUBCNL | H3K18la at RUBCNL promoter | | Autophagy induction and bevacizumab resistance via sustained VEGFA/ANGPTL4 transcription under hypoxia | CRC cell lines, xenografts and patient tumours treated with anti-VEGF | Confirmed in CRC | [33, 87] |
| Histone | H4K12la–GCLC | H4K12la at GCLC promoter | | Increases GCLC expression and glutathione synthesis, suppressing lipid peroxidation, ferroptosis and maintaining CRC stem-like cells | CRC stem-like cells and xenograft models | Confirmed in CRC | [88]. |
| Histone | H3K9la–GRAMD1A | H3K9la at GRAMD1A promoter | | KRAS-driven GRAMD1A expression, enhanced cholesterol transport, invasion and metastasis | KRAS-mutant CRC cell lines and mouse models | Confirmed in CRC | [93] |
| Histone | Global H3K18la/H4K12la | H3K18la and H4K12la (genome-wide) | | Associate with advanced stage, lymph-node metastasis, shorter disease-free survival and poor treatment response | CRC tissue cohorts | Confirmed in CRC | [90, 94] |
| Histone | H3K18la–EMT/invasion genes | H3K18la at SNAI1, ZEB1, VIM and related EMT loci | | Drives EMT and invasion; enriched at invasive fronts where stromal MCT4-dependent lactate sustains lactylation | CRC tissues and organoids; spatial profiling | Supported in CRC models | [83, 102] |
| Histone | H3K18la–immune checkpoints | H3K18la at B7-H3 and PD-L1 loci | | Upregulates immune checkpoints and promotes T-cell evasion | CRC cell lines and tumour samples | Supported in CRC models | [103, 104] |
| Histone | H3K18la in tumour-associated macrophages | H3K18la at RARγ/TRAF6–IL-6–STAT3 axis | | Induces M2-like polarisation and IL-6–STAT3 signaling; supports an immunosuppressive TME, especially in MSS CRC | CRC-associated macrophages (mouse and human) | Confirmed in CRC | [116, 117] |
| Histone | H3K18la in inflammatory macrophages | H3K18la at wound-healing genes (e.g. ARG1) | | Activates wound-healing and resolution programs; tolerogenic macrophage phenotype | Non-tumour inflammatory macrophage models | Extrapolated to CRC microenvironment | [15, 79] |
| Non-histone | eEF1A2 | K408la | | Enhances translational elongation and protein synthesis; promotes CRC cell growth under high lactate; reversed by KAT8 or MCT1/4 inhibition | CRC cell lines and xenografts | Supported in CRC models | [108] |
| Non-histone | NBS1 | K388la | | Stabilises MRN complex and homologous recombination; increases resistance to genotoxic stress | Non-CRC solid tumour models | Extrapolated to CRC | [111] |
| Non-histone | MRE11 | K673la | | Facilitates DNA end resection and high-fidelity repair; supports survival after DNA damage | Non-CRC solid tumour models | Extrapolated to CRC | [111] |
| Non-histone | TP53 (p53) | | K382la | Weakens DNA binding; suppresses pro-apoptotic targets; biases transcription towards glycolytic/antioxidant genes | Multiple non-CRC solid tumours | Extrapolated to CRC | [107] |
| Non-histone | YAP | | K90la | Enhances nuclear retention and TEAD-dependent transcription; reinforces proliferative and stress-adaptive programs | Hepatocellular carcinoma and other non-CRC tumours | Extrapolated to CRC | [112] |
| Non-histone | cGAS | | Multiple lysine sites | Attenuates cytosolic DNA sensing and type I interferon production | Immune and tumour models (non-CRC) | Speculative for CRC | [113] |
| Non-histone | HMGB1 | | Multiple lysine sites | Promotes neutrophil extracellular trap formation and pro-inflammatory signaling in acute injury | Acute kidney injury and sepsis models | Speculative for CRC | [114, 115] |
| Non-histone | HK2, PFKP, PKM2, LDHA | | Multiple lysine sites (lactylome-defined) | Modulate glycolytic enzyme activity and feedback on glycolytic flux while preserving lactate pools for signaling | CRC and colon cancer cell lines (lactylome studies) | Supported in CRC cell lines | [91, 92] |

Evidence levels: "Confirmed in CRC" = directly demonstrated in CRC models or patient samples; "Supported in CRC models" = shown in CRC cell lines or xenografts only; "Extrapolated to CRC" = demonstrated in other tumour types and mechanistically inferred for CRC; "Speculative for CRC" = not yet shown in CRC but biologically plausible.

This classification is intended to transparently distinguish evidence strength rather than imply equal levels of validation across tumour types.
